# Back to WHAT? The role of research ethics in pandemic times

**DOI:** 10.1007/s11019-020-09984-x

**Published:** 2020-11-03

**Authors:** Jan Helge Solbakk, Heidi Beate Bentzen, Søren Holm, Anne Kari Tolo Heggestad, Bjørn Hofmann, Annette Robertsen, Anne Hambro Alnæs, Shereen Cox, Reidar Pedersen, Rose Bernabe

**Affiliations:** 1 Faculty of Medicine, Center for Medical Ethics, Institute of Health and Society, University of Oslo, Blindern, Box 1130, 0318 Oslo, Norway; 2 Faculty of Law, Norwegian Research Center for Computers and Law, University of Oslo, Oslo, Norway; 3 Division of Emergencies and Critical Care, Department of Anaesthesiology, Oslo University Hospital, Oslo, Norway; 4 Department of Clinical Medicine, University of Oslo, Oslo, Norway; 5 Faculty of Health Studies, VID Specialized University, Oslo, Bergen, Stavanger and Sandnes, Norway; 6 Department of Health Sciences, The Norwegian University for Science and Technology, Gjøvik, Norway; 7 The Faculty of Health and Social Sciences, University of Southeastern Norway, Kongsberg, Norway; 8 Department of Law, School of Social Science, Centre for Social Ethics and Policy, University of Manchester, Manchester, UK

**Keywords:** Covid-19, Challenge studies, Exceptionalism, Research ethics, Uncertainty, Vulnerability

## Abstract

The Covid-19 pandemic creates an unprecedented threatening situation worldwide with an urgent need for critical reflection and new knowledge production, but also a need for imminent action despite prevailing knowledge gaps and multilevel uncertainty. With regard to the role of research ethics in these pandemic times some argue in favor of exceptionalism, others, including the authors of this paper, emphasize the urgent need to remain committed to core ethical principles and fundamental human rights obligations all reflected in research regulations and guidelines carefully crafted over time. In this paper we disentangle some of the arguments put forward in the ongoing debate about Covid-19 human challenge studies (CHIs) and the concomitant role of health-related research ethics in pandemic times. We suggest it might be helpful to think through a lens differentiating between risk, strict uncertainty and ignorance. We provide some examples of lessons learned by harm done in the name of research in the past and discuss the relevance of this legacy in the current situation.

## Introduction

These are uncertain times—for all, wherever one lives, and whatever one aspires to know about the Covid-19 pandemic. At present, there are so many things we do not (yet) know: how it will evolve and spread, when, if ever, it will end, how and why it started, whether infected persons develop permanent immunity, whether safe and effective cures and vaccines will be possible to develop, and last, but not least, what impact the pandemic will have on each and every one; be it individuals, families, societies, nations, regions, or globally. The aim of this paper is to address the ongoing debate about Covid-19 CHIs and discuss the concomitant role of medical and health-related research ethics in the present situation where there is an urgent need for developing effective methods of detection, treatment, and prevention to cope with the Covid-19 pandemic and notably in ways that minimize harm and that benefit all human beings. Although at present all attention and focus is on the Covid-19 pandemic, it is key not to forget there have been pandemic surprises in the past and that future, unknown pathogens may lead to crises with unprecedented implications. The issues we discuss here are therefore relevant beyond the Covid-19 pandemic; above all they concern how we should navigate (research ethics) in times of great threats to individual, public and population health and under conditions where different forms of uncertainty still prevail.^1^

The British Medical Association’s report on biomedical research and human rights states: «Research is driven by a desire to understand the causes of disease or dysfunction and find effective methods of prevention and treatment.”^2^ However, the report continues, “even such humanitarian aims can be risky”, in particular under circumstances perceived as extreme or exceptional.^3^ In the report, nine risk-factors for abusive research are identified, of which three are of particular relevance in the present context: (1) the perception of an urgent and overriding scientific need; (2) the perception of a national necessity or government pressure to conduct research; and (3) the situation of contingent populations chosen as research subjects.^4^

In a recent Letter to the Editor of the American Journal of Bioethics, Stoeklé and Hervé state that now is not the right time for ethics reflection, but rather for political action and for “indisputable confidence in medical care staff and scientists, not only in France, but everywhere around the world”.^5^ In a response to this view, and notably with the opposite title, the respondents state^6^:

In times of crisis, like the current pandemic of COVID-19, the perception that ethical standards can be relaxed due to the urgent need for solutions is growing, according to Stoeklé and Hervé. For them, ‘Ethics is only useful if you have the time, and right now, time is exactly what we do not have.’ It is a misperception without any doubts. Ethics has always preserved its identity as a rationalization of human action. Therefore, ethical reflections to take decisions are useful all the time and must be reinforced in times of pandemic.

Another way of visualising this tension is by differentiating between the *epistemological ethos* of doing biomedical research, such as developing knowledge and skills for effective diagnostic, treatment and prevention, versus the *ethical ethos* of the same enterprise; the attempt to protect the interests and wellbeing of patients and healthy individuals involved in such research. *Ethos* is here used in the meaning of ‘accepted standards.’ In research, two such normative standards or rules of play are used: *Epistemological* rules of play (such as truth, probability, coherence, relevance, fruitfulness, interestingness, and utility), and *ethical* rules of play (such as autonomy, informed consent, justice, beneficence, non-maleficence, truthfulness, dignity, trust, vulnerability, and solidarity). The tension between these two normative standards is permanent, and one that probably never can be fully resolved. But in times of perceived urgency, the danger is increased tension, or worse, a disregard and violation of epistemological as well as ethical standards, and that short-sighted expediency with questionable results will ensue.

In the pages to follow we argue in favour of an ethics of precaution, with particular emphasis on the role of research ethics in particularly challenging situations, such as pandemics. That is, we will analyse and critically assess the epistemological and ethical justification of several research initiatives that have been implemented or are at the planning stage. We claim that in the current situation of a palpable sense of medical and scientific urgency, and of national and global necessity, there should be no room for epistemological or ethical exceptions or shortcuts. On the contrary—and perhaps more than ever—there is a need to conduct biomedical and health-related research in compliance with existing rules of play and fundamental human rights commitments.

## Three forms of uncertainty

Faced with the “toxic brew of uncertainty”^7^ the pandemic has caused, we suggest it would be helpful to differentiate between three different forms of uncertainty: *Risk*, *strict uncertainty*, and *ignorance or non-knowledge*. Risk represents a form of uncertainty with known potential outcomes, and, where the probability distribution is known. The plethora of uncertainties that the Covid-19 pandemic are causing cannot, however, be addressed within this narrow framework of risk estimation; uncertainty considerations of the two additional kinds mentioned above should also be included. S*trict* or *fundamental* uncertainty is a form of uncertainty where possible outcomes are known, but the probability distribution is unknown, while *ignorance or non-knowledge* represents forms of uncertainty where only some possible outcomes are known while the statistical likelihood of each of them is unknown.^8^ The relevance of differentiating between these three forms of uncertainty in the present context is visualized in Table 1.

## The disaster that taught the world why ethics and human rights matter

In the aftermath of another global disaster, World War II, several normative initiatives were taken to prevent a similar catastrophe to recur, such as the establishment of the United Nations and the development of the Universal Declaration of Human Rights. In addition, more robust ethical standards for biomedical research were crafted in order to avoid inhuman research in the future—not the least in crisis-situations. The then First Lady of the USA, Eleanor Roosevelt, served as the first Chair of the UN Commission on Human Rights, which drafted the Universal Declaration of Human Rights (1948). The first paragraph in the Declaration’s Preamble states that “the foundation of freedom, justice, and peace in the world” is the “recognition of the inherent dignity and of the equal and inalienable rights of all members of the human family”.^9^ Of the 30 Articles in the Declaration Article 1 and 5 are of particular relevance for research ethics; Article 1 restates the freedom and equality of all human beings in terms of dignity and rights, and Article 5 emphasizes the right to be protected from torture or cruel, inhuman or degrading treatment or punishment. The essence of these normative statements harkens back to the ethical code of medical research issued by the war crimes tribunal at Nuremberg the previous year, in 1947.^10^ Of the 10 standards laid down in this Code, and with which physician-researchers must comply when carrying out experiments on human subjects, standard 5, in particular, has become highly relevant these days due to pressure from influential medical stakeholders, agencies and bioethicists to permit the conduct of *controlled human infection studies* (CHIs), also labeled *human challenge trials* (HCTs), or *challenge studies* (CSs) to possibly shorten the development time of vaccines to protect against Covid-19 caused by the SARS-CoV-2 virus.^11^ In the next paragraph of this paper we will examine and critique in detail four position statements advocating the epistemological and ethical justifiability of conducting Covid-19 CHI-studies: P. Singer and R.Y. Chappell’s, *Pandemic ethics: The case for experiments on human volunteers*^12^; the report, *Key criteria for the ethical acceptability of COVID-19 human challenge studies*, issued by a working group set up by WHO^13^; the Policy Forum statement, *Ethics of controlled human infection to study COVID-19, by* Shah, Miller, Darton et al.;^14^ and Jamrozik and Selgelid’s statement, *COVID-19 human challenge studies: ethical issues*.^15^ In addition, we will consider other recent papers advocating the use of Covid-19 CHIs studies. In Table 2 below we have made a summary of prevalent arguments for and against such studies.

**Table 2:** Summary of arguments for and against CHIs and Covid-19 CHIs

Type of argument	Pro	Con
The acceleration and shorter time argument	CHIs can substantially accelerate testing—and widespread rollout^a^ Results come much faster with HCIs than with phase III vaccine trials^b^	The development of a robust challenge model for testing SARSCoV-2 vaccines may be 1–2 years. Given that SARS-CoV-2 vaccines will enter phase 3 trials imminently, these scientific and technical factors alone make CHIs unlikely to accelerate the establishment of vaccine efficacy^c^
The low cost argument^d^	CHIs are less expensive than vaccine trials	
The scientific merit argument^e^	CHIs can be used to clarify dynamics of infection, viral pathogenesis, and risk of vaccine pathogenesis	A model of disease in healthy young volunteers may have questionable scientific validity when extrapolated to older or other at-risk populations that have disproportionate morbidity^f^
The controlled environment argument^g^	CHIs are conducted in a controlled environment, making it easier for researchers to study the natural progression of the disease than it would be in the field	
The fewer trial participant argument^h^	CHIs require much fewer research participants	
The exceptionalist argument	Extraordinary diseases require extraordinary solutions^i^ The urgency of the current pandemic gives substantial weight to a challenge study^j^	
The endemic argument	The probability of dying or developing disability if infected would be smaller in a CHI trial^k^ The risks in question do not entail a major net increase in risk (in light of background risks of infection)^l^ Participants face a background risk of infection in the community^m^ Only people with an especially high baseline risk of getting exposed during or soon after the trial period should be recruited (e.g., people residing in areas with high transmission rates)^n^	
The risk and safety argument	Participation in a Covid 19-CHI trial would be less risky than joining a standard efficacy trial for the same vaccine^o^	Covid-19 CHIs have a much higher risk than the minor risk threshold.^p^ For a live SARS-CoV-2 challenge there are deadly risks^q^ Currently, we lack sufficient knowledge of SARSCoV-2 pathogenesis to inform inclusion and exclusion criteria for a SARS-CoV-2 CHIM^r^
The medical benefit argument	The probability of averting death in the event of infection would be substantially better inside a CHI trial than outside^s^	
The altruistic argument	It might seem that anybody volunteering to participate in such a study lacks capacity for rational decision-making. But humans do many important things out of altruism^t^ Volunteers who participate in the challenge trials should be motivated to advance human health and wellbeing rather than driven by their economic needs^u^	
The social value argument	Benefits to the subject + benefits to society > risks to the subject^v,w^ Given the risks to participants, SARS-CoV-2 challenge studies would need to demonstrate very substantial social value before proceeding. Arguably, this bar might already be met given the high death toll and severe disruption caused by the pandemic^x^ Covid-19 CHIs could help prioritize among the almost 100 investigational vaccines and over 100 experimental treatments for COVID-19 currently in development^y^	Young healthy adults may not generalize to older individuals and those with comorbidities who would most benefit from effective vaccines^z^

^a^Eyal (2020) and Callaway (2020)

^b^The International Alliance for Biological Standardization (2019), Jamrozik and Selgelid (2020a, b), Menikoff (2020), Eyal et al. (2020, pp. 1753–1754)

^c^Deming et al. (2020)

^d^The International Alliance for Biological Standardization (2019), Jamrozik and Selgelid (2020a)

^e^Bambery et al. (2020, pp. 93–94), Shah et al. (2017, pp. 20–22), Jamrozik and Selgelid (2020b, pp. 602–603), Shah et al. (2020, p. 2) and Wolemonwu (2020, p. 2)

^f^Deming et al. (2020)

^g^Schaefer et al. (2020)

^h^The International Alliance for Biological Standardization (2019), Jamrozik and Selgelid (2020a)

^i^Stoeklé and Hérve (2020), Plotkin and Caplan (2020)

^j^Schaefer et al. (2020)

^k^Eyal (2020, p. 26)

^l^Jamrozik and Selgelid (2020a, p. 3)

^m^WHO (2020, p. 9)

^n^Eyal et al. (2020, pp. 1754–1755)

^o^Eyal (2020, p. 25)

^p^WHO (2020, p. 10)

^q^Eyal (2020, p. 24)

^r^Deming et al. (2020)

^s^Eyal (2020, p. 27)

^t^Callaway (2020)

^u^Wolemonwu (2020)

^v^Menikoff (2020, p. 81)

^w^Eyal et al. (2020, p. 1754)

^x^Schaefer et al. (2020)

^y^Shah et al. (2020, p. 2)

^z^Schaefer et al. (2020)

Standard 5 of the Nuremberg Code reads:

No experiment should be conducted where there is an a priori reason to believe that death or disabling injury will occur; except, perhaps, in those experiments where the experimental physicians also serve as subjects.^16^

While repeating (in the first clause of Article 7) that no one shall be subjected to torture or to cruel, inhumane or degrading treatment or punishment, the legally binding 1966 UN International Covenant on Civil and Political Rights adds a second clause that “In particular, no one shall be subjected without his free consent to medical or scientific experimentation”.^17^ The prohibition is intended to prevent the recurrence of atrocities such as those that took place during WWII and the first decades after the war. Even though this prohibition is implicit in the first clause, the matter was deemed so important as to require a specific and precise provision.^18^ During the drafting of the Covenant, there was a proposal that compulsory measures might be taken “in the interest of community health”, but the proposal was rejected on the grounds that this might lead to abuse.^19^ The consent requirement is thus formulated as absolute, without any exceptions. The Human Rights Committee has reaffirmed what is already explicitly mentioned in Article 4 (2), that even in situations of public emergency, no derogation from Article 7 is allowed.^20^

## Are controlled human infection studies (CHIs) with SARS-CoV-2 justified?

Controlled Human Infection studies (CHIs) “are clinical studies that, as part of the protocol, deliberately expose trial participants to an infectious pathogen. These studies are often done in the context of vaccine development, with trial candidates exposed to a pathogen after being immunized with an experimental vaccine”.^21^ The main advantages of CHIs compared to large field trials are that they can generate data much faster, they are much less expensive and they do not require thousands of research participants, normally only between 10 and 50 participants.^22^ The four position statements on CHIs referred to above all suggest that suitable candidates for such studies are “young people without underlying medical conditions”^23^/“healthy young volunteers”^24^/“young healthy adults”.^25^ The last part of Standard 5 of the Nuremberg Code deserves particular attention in this context because it rejects high risk experiments with human beings be they young or old, “except perhaps”, in cases where physician researchers are willing to conduct such experiments on themselves.

The proponents of starting such studies all admit that there remains “significant uncertainty” with regard to the pathogenic effects of SARS-CoV-2 in both elderly and young people, and that high or significant risk is involved in such studies with the potential of causing severe harm, or death.^26^ Even if the recommendations of the Nuremberg Code of letting researchers themselves serve as participants had been followed strong caution is still warranted for at least six reasons. First, as uncertainty still surrounds the pathogenic effect of the SARS-CoV-2 in different age groups, challenge-studies would not be justified because of the lack of robust knowledge and understanding of the short term consequences of the SARS-CoV-2,^27^ and because of the almost complete absence of data on the long term effects of the virus.^28^ For these reasons it would be very difficult to justify the inclusion even of the most knowledgeable individuals in such studies, i.e. Covid-19 researchers, because even the most knowledgeable are currently deeply ignorant.^29^ It is worth noting here that in a document on challenge-studies for vaccine development adopted in 2016 by WHO’s Expert Committee on Biological Standardization there is no talk about accepting such studies if the risk is of the size referred to in the WHO-working group’s report of this year; i.e. “high” risk with the potential of causing “severe harm”.^30^ On the contrary, in the 2016 document emphasis is on “minimizing risks to subjects” and reference is made to “situations in which there may be greater-than-minimal risk”, or risks “…considerably greater than minimal”, but still manageable as “…e.g. accepting that they [the trial candidates] will develop an acute, but manageable, disease that will resolve but in the meantime may cause considerable morbidity, such as severe diarrhea managed with fluid and electrolyte replacement”.^31^ However, risks “considerably greater than minimal” are still far from being equal to high risk with the potential of causing severe harm. Further down the same paragraph of the 2016 WHO Expert Committee document the following is added: “However, accepting such risks requires absolutely that the elements of voluntary consent are based on truly being informed”.^32^ It is, however, difficult to see how it would be possible to comply with this condition when so much is still unknown with regard to both the short and long-term pathogenic effects of the SARS-CoV-2. A valid informed consent is a legally binding absolute human rights requirement. Second, if something goes wrong after infecting healthy young volunteers with the virus, the treatment options at present are limited. Accepting such studies in relation to the SARS-CoV-2 would therefore not be in compliance with two of the core conditions in current ethical frameworks for challenge studies: namely that the pathogen studied does not induce infections that may cause severe harm, or for which there exist no effective treatment.^33^ Despite this, challenge studies for Covid-19 are proceeding and the recruitment of health volunteers has exceeded 30,000.^34^ If something goes wrong, compensation to participants ought to be a major consideration for these trials. In a recent publication, Carl Elliot argues that although the focus of challenge studies is to recruit healthy volunteers, if the model is based on remunerating these volunteers, the studies may attract economically vulnerable volunteers who view participation as a means to an end.^35^ And as stated by Ruth Macklin^36^:

…given standard practice, it is virtually certain that monetary payment—which may be considerable—will serve as an inducement to enroll. The likely result is that a disproportionate number of volunteers would come from lower-income brackets, including many people who lost their jobs because of the pandemic. It is also likely that many volunteers would be members of racial and ethnic minorities, raising a serious question of social justice.

Economically vulnerable volunteers who become injured or “permanently disabled” may not receive compensation, especially in the US.^37^ In contrast to other developed countries, research sponsors in the US are not under any legal obligation to pay for the medical care of research participants who become injured or ill, hence, few do.^38^ Furthermore, the US National Vaccine Injury Compensation Program excludes payment for experimental vaccines.^39^ Research participants in the US can therefore be left without compensation for injury caused by their participation in a challenge study. Third, the technical label ‘*controlled* human infection studies’ is unfortunate, because it might give healthy young volunteers and other non-experts the impression of a kind of control that is misleading. Fourth, at present one does not know whether controlled infections studies conducted with young and healthy adults (or COVID19-researchers) will provide results that enhance survival and/or healing-rates of those most affected by SARS-CoV-2, i.e. old fragile persons with comorbidities.^40^ Hence, there is a significant problem of external validity. Fifth, the argument that participants “might benefit from controlled infection and/or vaccination if they become immune to virus”^41^ is also undermined by major uncertainty, as openly admitted by the proponents of such studies.^42^ Sixth, and, perhaps, most important, such studies violate the core ethical principle of human subjects research; i.e. the *priority of the individual* principle. This principle emerged as a normative response to the medical horrors that had been practiced during WWII in concentration camps in Nazi Germany and in Japan. Yet in spite of the Universal Declaration of Human Rights and the Nuremberg Code, several ethically compromised studies involving vulnerable groups have been conducted after WWII.

To underscore this point, and analyze their implications, allow us to describe two such examples. First, the hepatitis studies, conducted from 1953, at Willowbrook State School, an institution for mentally disabled children on Staten Island, New York. More than 700 children, predominantly African-American and Puerto-Rican, were included in these studies, and a subgroup of almost 100 non-infected children were fed a suspension with the local strains of the hepatitis virus prepared from the stool of six children collected “during the first 8 days of recognized jaundice”.^43^ Written consent from the parents of these children had been obtained by the chief investigator, Dr. Krugman. But later investigations indicated that the parents’ consent might have been based on indirect coercion since volunteering their children to the infection study was allegedly put forward as a condition for admitting the children to care at Willowbrook State School. Krugman defended the contested infection and immunization studies his whole life with reference to their scientific merits; the “confirmation of two types of hepatitis, A and B, with different infection pathways (oral versus close contact), and the preparation of a “crude vaccine” containing the hepatitis B virus.^44^ In a Letter to the Editor of the Lancet in 1971 he justified the exposure of “a small number of newly admitted children to the Willowbrook strains” of the hepatitis virus with reference to: (1) *inevitability*; the children were “bound to be exposed” to the same virus strains “under the natural conditions existing in the institution”, (2) *safety*; they would be admitted to “a special, well-equipped and well-staffed unit”, thus shielding them from exposure to other prevalent infectious diseases in the institution, (3) *immunity*; “they were likely to have a subclinical infection followed by immunity”, and (4) *informed consent;* “only children with parents who gave their informed consent would be included”.^45^

The second study worth mentioning is the cancer injection study that took place in the early 1960s at the Jewish Chronic Disease Hospital in Brooklyn, New York. The chief investigator, Dr. Chester M. Southam, had since 1953 conducted research on the role of the immune system in protecting against cancer, using two different groups of research participants who were injected with a suspension of foreign cancer cells to study the difference in immunological rejection of the cells between the two groups. The first group was a cohort of patients at Memorial Hospital in Ohio, in total 300, with different forms of widespread cancer, and the second group consisted of 300 healthy individuals from the Ohio State Penitentiary^46^; i.e. a prison in downtown Columbus, Ohio. These studies documented that healthy individuals rejected the injected cancers cells faster than the cancer patients (4–6 weeks versus 6 weeks to 3 months).^47^ In his presentation of Southam’s studies John D. Arras labeled the second group of research subjects “healthy prison volunteers”, and notably, without any reflection whatsoever either about the ethical justifiability of recruiting prisoners for a study that would not benefit them, or whether it is appropriate to consider prisoners to be *free* to volunteer.^48^ On this point Southam himself seems to have been, at least, partly aware of the dilemma of recruiting incarcerated individuals. In an interview in Science he admitted that, although there was no theoretical likelihood that the injections would produce cancer, he had nonetheless been unwilling to inject himself, or his colleagues, when there was a group of normal volunteers at the Ohio Penitentiary fully informed about the experiment and its possible risks and nonetheless eager to take part in it:

“I would not have hesitated”, Southam said, “if it had served a useful purpose. But to me it seemed like false heroism, like the old question whether the General should march behind or in front of his troops. I do not regard myself indispensable—if I were not doing this work someone else would be—and I did not regard the experiment as dangerous. But, let’s face it, there are relatively few cancer researchers, and it seemed stupid to take even the little risk”.^49^

Southam persuaded the then director of the Jewish Chronic Disease Hospital, Emmanuel E. Mandel, to permit, as a third part of his immune reaction studies, the injection of foreign cancer cells into 22 old patients with other debilitating chronic diseases than cancer. The scientific justification for this study was to get “direct evidence” that it was the cancer disease that caused the delay in rejection of the foreign cancer cells, and not the fact that most of the cancer patients he had studied were elderly, debilitated and with additional chronic diseases.^50^ Such evidence, he maintained, would be possible to establish by doing the same immune reaction study in a group of elderly patients with other chronic and debilitating diseases than cancer. In the letter Southam wrote to Mandel on July 5, 1964, he also discussed whether consent (“written permission”) from the patients was warranted, something which he warned against, for two reasons; first, at Memorial Hospital they considered it a “routine study, much less hazardous than other routine procedures”, and second, the only risk related to the use of cancer cells in these injections was the “phobia and ignorance surrounding the word “cancer”.^51^ In the same letter Southam informed Mandel that with regard to the 300 prisoners, “signed permits” had been obtained, but this, according to Southam, was “because of the law oriented personalities of these men, rather than for any medical reason”.^52^ In 1966 the two doctors were found guilty of fraud, deceit and unprofessional conduct, and they were, in particular, criticized for assuming they were entitled to perform any kind of research without consent as long as the research in question was scientifically justified. Two years later, however, Southam, was elected President of the American Association for Cancer Research for the period of 1968–1969.

In 2001 Miller and Grady proposed a way of evaluating the ethical justifiability of planned challenge-studies by locating each candidate along a continuum from legitimate studies to clearly unacceptable ones. In the “border zone” between these two extremes they locate studies that are neither indisputably justifiable nor clearly unacceptable. Among legitimate ones they count studies for common cold, cholera^53^ and malaria,^54^ while CHI-models for Lyme disease or Helicobacter pylori are labeled “more controversial”.^55^ Finally, among clearly unacceptable studies they mention two examples; HIV-CHI-studies or CHI-studies for hepatitis C virus.^56^ Their arguments for labeling these two CHI-models unacceptable were twofold; “non-existent or ineffective” treatment, and “intolerable symptoms and/or the likelihood of serious morbidity or mortality”.^57^ Two additional possible studies deemed unacceptable are referred to in CIOMS’ commentary on guideline 4 of the International Ethical Guidelines for Health-related Research involving Humans^58^:

For example, a study that involves deliberately infecting healthy individuals with anthrax or Ebola—both of which pose a very high mortality risk due to the absence of effective treatments—would not be acceptable even if it could result in developing an effective vaccine against these diseases. Therefore, researchers, sponsors, and research ethics committees must ensure that the risks are reasonable in light of the social and scientific value of the research, and that the study does not exceed an upper limit of risks to study participants.

An additional example—deemed unacceptable at the time of the evaluation—was a Zika virus-CHI-study.^59^ The NIH ethical review committee’s reasons for deciding against the study were with reference to three kinds of uncertainty; uncertainty of the risk to research participants as well as to third parties (fetus and sexual contacts), uncertainty about the duration of protection needed, and, third, uncertainty of the study’s societal value.^60^

Viewing the studies conducted by Drs. Krugman and Southam in the light of Miller and Grady’s differentiation between legitimate, more controversial and clearly unacceptable challenge-studies, and in view of current ethical standards pertaining to such studies, calls for reflection, not only for historical reasons; they may also be of help in investigating where the arguments in favor of CHI-studies in the current context of the Covid-19-pandemic differ from those of Krugman and Southam, and where these arguments seem to overlap. When it comes to locating the studies under discussion along the ethical line of decreasing permissibility proposed by Miller and Grady, Southam’s immune reaction studies on elderly, debilitated subjects arguably deserve the label ethically unacceptable, in spite of their scientific merits, for at least three reasons: (a) no consent was obtained, (b) the element of deceit involved and (c) the fact that participants did not stand to benefit in any ways from the studies. Southam’s reluctance against using himself and colleagues as study subjects was based on two considerations: first, exposure to possible risks caused by the injection of foreign cancer cells; second, since neither he nor his colleagues were physically debilitated they were unsuitable candidates for the study. In two of the position statements referred to above reservation is expressed against using researchers as study-subjects in Covid-19 CIH-studies, although for a different reason; “clinical and research staff might feel pressure to participate”,^61^ and “such individuals could feel pressured to participate (thereby undermining the voluntariness of informed consent”.^62^

Krugman’s challenge studies at Willowbrook are more difficult to locate and label according to Miller and Grady’s linear differentiation, not least because his defense of the studies has several striking similarities with arguments used in favor of conducting CHI-studies to speed up the development of efficient vaccines against the SARS-CoV-2 virus. First the reference to the studies’ *scientific merit*; the “confirmation of two types of hepatitis, A and B, with different infection pathways, and the preparation of a “crude vaccine” containing the hepatitis B virus.^63^ A similar type of argument is used by And Shah, Miller, Darton et al.^64^:

For example, CHIs could clarify dynamics of infection, viral pathogenesis, and risk of vaccine pathogenesis or identify correlates of protection—all of which could inform the development and implementation of vaccines.

Second, their *high social value*. In his Letter to the Editor of the Lancet in 1971 Krugman emphasized the high social value beyond Willowbrook of his studies: “It is unnecessary to point out the additional benefit to the world-wide population which have been plagued by an insoluble hepatitis problem for many generations”.^65^ In support of such arguments, Shah, Miller, Darton et al. argue^66^:

SARS-CoV-2 CHIs could have high social value in several ways. For example, they could help prioritize among the almost 100 investigational vaccines and over 100 experimental treatments for COVID-19 currently in development. CHIs could help identify the most promising agents, which would inform the design of larger trials, guide decisions to scale up manufacturing early, and thereby accelerate product development and implementation.

In a similar vein, Singer and Chappel refer to the high social value, or in their wording—“the broader humanitarian benefits”—of such studies.^67^ Third, *the exposure of a small number of individuals to risk for the sake of benefits to the rest.* Different versions of this argument are used in the four position statements,^68^ and also by the International Alliance for Biological Standardization.^69^ Fourth, *the endemic argument*—the children were “bound to be exposed” to the same virus strains “under the natural conditions existing in the institution”.^70^ Two 2020-versions of the same argument read: “the risks in question do not entail a major net increase in risk (in light of background risks of infection”,^71^ and “participants face a background risk of infection in the community”.^72^ Fifth, the *safety*- and *better care* argument. In Krugman’s wording the study subjects would be admitted to “a special, well-equipped and well-staffed unit”,^73^ while Shah, Miller, Darton et al. deem long-term follow-up *critical*^74^:

To minimize risks to study personnel, participants should be in inpatient isolation, with contact reduced to the extent possible and robust personal protective equipment provided.

And WHO’s working group highlights the importance of “supportive care, including critical care” and “long-term follow-up” as two crucial risk-minimization strategies.^75^ Sixth, the *immunity-*argument; Krugman argued that the children involved in his studies “were likely to have a subclinical infection followed by immunity”^76^, while WHO’s working group refers to “[i]mmunity induced by experimental vaccines” as a potential benefit of study participants.^77^ Jamrozik and Selgelid combine the safety-/better care argument with the immunity argument^78^:

…potential direct benefits of being infected with SARS-CoV-2 in the course of human challenge studies would include participants being exposed to less infection-related risk than if they are infected in the community (e.g. because of early diagnosis and medical care) and gaining immunity to future infection in the context of a high background risk.

Seventh, and last but not least, Krugman as well as the authors of the four position statements referred to above all emphasize the importance of *informed consent.*^79^

This comparison between Krugman’s arguments and the arguments in the four position statements here subject of detailed analysis and in other recent papers advocating the use of Covid-19 CHIs shows that their overall views are pretty much the same. In fact, the only difference in terms of substance is the use of vulnerable individuals or groups as study-participants which two of the four position statements warn against.^80^ Singer and Chappel do not address this issue, while the authors behind the fourth position statement, Jamrozik and Selgelid, in a recent paper on CIH studies in endemic settings, argue that sometimes it may be justifiable, in fact “ethically important” to include vulnerable populations: “…especially where the results of research in other populations are not likely to be generalisable to the vulnerable populations in question. This is one consideration that sometimes favours conducting (more) HCS in low-middle income countries (LMICs)”.^81^

So where does all this lead us? Should we accept the ‘neo-Krugman’ian’ views advocated by proponents of Covid-19 CHIs, or should we rely on the normative principles that were formulated as a reaction to the kind of studies Southam and Krugman had conducted? Of these principles the *priority of the individual* principle is the most fundamental. The original formulation of this principle in biomedical research ethics occurred in the first, i.e the 1964-version of the Declaration of Helsinki as Basic Principle 5 and reads^82^:

Every biomedical research project involving human subjects should be preceded by careful assessment of predictable risks in comparison with foreseeable benefits to the subject or to others. Concern for the interests of the subject must always prevail over the interests of science and society.

This principle has been maintained in all versions of the Declaration. In the latest version (WMA 2013) it is included as General Principle 8:

While the primary purpose of medical research is to generate new knowledge, this goal can never take precedence over the rights and interests of individual research subjects.

Whereas the Declaration of Helsinki is a professional ethics norm, the principle was restated in Article 3 of the UNESCO Universal Declaration on Bioethics and Human Rights, which was adopted by all Member States of the United Nations in October 2005, and notably with reference in the first section of the article to human dignity, human rights and fundamental freedoms:

The interests and welfare of the individual should have priority over the sole interest of science or society.

In their position statement Singer and Chappell claim that current research-ethical principles are based on “assumptions developed in calmer times when much less was at stake”.^83^ This claim is historically wrong, as this is not the first time the world has faced a public health crisis since the development of the core principles of research ethics. We have managed to live through those less calm times without infringing those principles, and we have no particular reason to overstep them now.

So to conclude our analysis and critique of controlled human infection studies (CHIs) with SARS-CoV-2: There is a consistent, historical line of research ethical principle from Nuremberg, through the revisions of the Helsinki Declaration, and the successive UN human rights declarations and other normative documents. This principle is that the interests of the individual research participant is paramount in research ethics, i.e. that if there is a conflict between the interests of society (e.g. in speeding up vaccine development) and the interests of the participants (e.g. in not dying or being permanently harmed) then the interests of society has to yield to the interests of the participant. There is no exception for times of crisis, or for instances where societal interests are large.

One might argue against this principle, and some have done so, but well-founded and sustainable ethical principles can’t be disregarded just because they seem inconvenient at a certain point in time. The concern here is not just an abstract philosophical principle, but a part of the normative core of all existing research ethics processes. If we take this principle seriously it prohibits the conduct of SARS-CoV-2 challenge studies at the present time where the challenge virus would be the native virus with full virulence and where there is no rescue treatment yet available.

## Against epistemological and ethical research exceptionalism

In a Policy Forum statement in Science Alex London and Jonathan Kimmelman, warn against “pandemic research exceptionalism” and against using crises as an excuse for lowering scientific standards.^84^ They focus on five epistemological conditions of “informativeness and social value” that research should embody, even in times of emergency: First, *importance*^85^:

Trials should address key evidence gaps…As of this writing, more than 18 clinical trials enrolling more than 75,000 patients have been registered in North America for testing various hydroxychloroquine regimens for COVID-19. This massive commitment concentrates resources on nearly identical clinical hypotheses, creates competition for recruitment, and neglects opportunities to test other clinical hypotheses.

Second, *rigorous design*. Third, *analytical integrity*: “Designs should be pre-specified in protocols, prospectively registered, and analyzed in accordance with pre-specification”. Fourth, *complete reporting*: “trials should be reported completely, promptly, and consistently with pre-specified analyses”. Fifth, and last, *feasibility*: “Studies must have a credible prospect of reaching their recruitment target and being completed within a time frame where the evidence is still actionable”.^86^

Similar warnings against research exceptionalism, and notably with reference to things that went wrong in the past when arguments in favor of this were used, are made by Deborah Doroshow, Scott Podolsky and Justin Barr^87^:

The coronavirus disease 2019 (COVID-19) pandemic has incited remarkable disruption in biomedical research. At academic institutions worldwide, laboratories have been forced to halt all but the most critical activities. Clinical trials of novel agents for such diseases as cancer are temporarily suspended, limiting access to potentially life-prolonging medications […] Although this boom has already begun to transform our response to the pandemic for the better, medical and scientific responses to past crises suggest that urgency may also result in compromised research quality and ethics, which may in turn jeopardize public faith in government and science, waste precious resources, and lead to the loss of human life.

## Epistemic shortcuts and ethical pitfalls

Another problem with knowledge-production of the Covid-19 pandemic has been the lowered standards of quality assurance of published research. It has been documented that the peer review process has been rushed (“express” or “opinion based peer review”)^88^ and so far (October 28, 2020) 37 research papers about Covid-19 have been retracted.^89^ A stunning example of this is an observational study based on the health records of almost 100,000 patients around the world published in the prestigious journal Lancet in May 2020, which indicated that hydroxy-chloroquine had a sharply higher risk of death and heart problems compared to those who did not receive the drug, and that hydroxy-chloroquine did not provide any benefit.^90^ On June 4, 2020 this study was retracted by the authors due to doubts about the veracity of the data used and the analyses conducted.^91^ On June 16, 2020 preliminary clinical trial results from the *Recovery study* of the University of Oxford were broadcasted all over the world suggesting that a commonly used drug—dexamethasone—reduced deaths among the sickest COVID-19 patients by a third.^92^Hopefully, the published study will prove this claim to be justified, but it is unfortunate when Covid-19 researchers start “doing science by press release”^93^ instead of following generally accepted publication procedures. As stated by Dr. Atul Gawande at Brigham and Women’s Hospital in Boston^94^:

Typically, researchers extensively detail their work in scientific journal articles. Before publication, other scientists take an in-depth look at how the study was designed, who the patients were and whether any potential side effects were uncovered—a process called peer review. It takes time—weeks or months in some cases—for independent, unbiased experts to pore over the manuscripts, looking for any concerns.

Taking epistemic shortcuts inevitably produces poor evidence and often leads to bad decisions with potentially severe consequences for vulnerable persons. Although tempting, we should avoid epistemic shortcuts, as high-quality evidence is needed in exceptional times, as otherwise. When taking chances, we must consider the risks of harm, not only the benefits.

One such risk worth mentioning here is that persons selected for testing emergent vaccines become victims of *enhanced disease*, i.e. presenting worse symptoms from the effects of an unproven vaccine compared to persons catching e.g. the Covid-19 flu through usual paths of contagion. Potential Covid-19 vaccine volunteers might e.g. end up with life-threatening complications (such as irreversible and untreatable clogged lungs) whereas, in the current situation most *un*vaccinated patients display only mild flu-indicators, if infected. As there as yet does not exist any known therapy, the perils these volunteers risk is ethically unacceptable.^95^ Two examples of enhanced disease precipitated by insufficiently proven vaccines occurred in connection with the inoculation of children against RSV (respiratory syncytial virus in the late 1960s.^96^ Similarly, from October 1976 to January 1977 more than 40 million adult citizens in the USA were vaccinated with a swine influenza virus vaccine. During the same period more than 500 vaccinated persons fell ill with a rare neurological illness (Guillain-Barré syndrome) and 25 of them died.^97^ These unexpected events led to immediate cancellation of the vaccination program indicating that the effects of an insufficiently tested vaccine in some cases cause greater harm than benefit. Hence, ethical reflection and compliance with epistemological rules of play are needed more than ever. In a paper reflecting on what might be learned from the 1976 Swine Flu Vaccination Program, Sencer and Millar warn against politicization of scientific information in a way that is well worth listening to also for today’s public health leaders and their political peers^98^:

While all decisions related to NIIP [the National Influenza Immunization Program] had been reached in public sessions (publishing of the initial virus findings in CDC's weekly newsletter, the Morbidity and Mortality Weekly Report (MMWR); New York Times reporter Harold Schmeck's coverage of the ACIP [the Advisory Committee on Immunization Practices of the United States Public Health Service] sessions, the President's press conference, and 4 congressional hearings), effective communication from scientifically qualified persons was lacking, and the perception prevailed that the program was motivated by politics rather than science. In retrospect (and to some observers at the time), the president's highly visible convened meeting and subsequent press conference, which included pictures of him being immunized, were mistakes. These instances seemed to underline the suspicion that the program was politically motivated, rather than a public health response to a possible catastrophe.

Annex 11 of the draft DHEW [Department of Health Education and Welfare] pandemic preparedness plan states, ‘For policy decisions and in communication, making clear what is not known is as important as stating what is known. When assumptions are made, the basis for the assumptions and the uncertainties surrounding them should be communicated’.^99^ This goal is much better accomplished if the explanations are communicated by those closest to the problem, who can give authoritative scientific information. Scientific information coming from a nonscientific political figure is likely to encourage skepticism, not enthusiasm.

## Research ethics and compassionate use

Another function of research ethics is to provide guidance on the “compassionate use” of drugs unapproved for Covid-19. In the past months we have read about compassionate use access of Covid-19 patients to hydroxychloroquine and remdesivir.^100^ By compassionate use (otherwise known as expanded access), we refer to^101^:

…a potential pathway for a patient with an immediately life-threatening condition or serious disease or condition to gain access to an investigational medical product (drug, biologic, or medical device) for treatment outside of clinical trials when no comparable or satisfactory alternative therapy options are available.

In principle compassionate use must have regulatory oversight and, in some countries such as the US, Spain, and Italy, research ethics committee approval as well.^102^ US regulations as well as those of individual EU member states stipulate the requirements before an investigational drug can be offered to either an individual, a limited group, or a wider population.^103^ But what exactly is the role of research ethics? Compassionate use is therapy in the sense that the purpose of it is to cure. However, compassionate use is not only therapy.^104^ It is, after all, the provision of a drug with yet-to-be determined levels of efficacy and safety. Considering the not very impressive success rate of an investigational drug of only 14% (i.e., from phase 1 to successfully being licensed for market distribution),^105^ the risks of compassionate use, especially when doctors cannot be provided with definitive guidance on dosage or exclusion criteria, can actually be worse than the risks of a controlled clinical trial. This being the case, research ethics guidance is imperative for access to investigational drugs via compassionate use. Also, faced with a surge of sick patients suffering from a new unknown disease, well-motivated clinicians and investigators all over the world, including drug companies and funding agencies, should adopt “a more integrated approach to learning while doing”, and they should join forces, i.e. engage in collaborative efforts so as to increase the “exploitation/exploration trade-offs”, and, hopefully, shorten “the period until effective treatments are discovered and implemented”.^106^

## Research ethics and post-trial access

One of the tenets of research ethics is the provision of the benefits of research to the intended patient population. All major international ethics guidelines for research have provisions with different degrees of specifications. The Declaration of Helsinki article 34, for example, requires clinical trial “sponsors, researchers, and host country governments” to ensure post-trial provisions to participants who, at the end of the trial, might still need the trial intervention that has been “identified as beneficial in the trial”.^107^ UNESCO’s Universal Declaration on Bioethics and Human Rights provides more specificity and directive. Article 15 says the following^108^:

Benefits resulting from any scientific research and its applications should be shared with society as a whole and within the international community, in particular with developing countries. In giving effect to this principle, benefits may take any of the following forms: (a) special and sustainable assistance to, and acknowledgement of, the persons and groups that have taken part in the research; (b) access to quality health care; (c) provision of new diagnostic and therapeutic modalities or products stemming from research; (d) support for health services; (e) access to scientific and technological knowledge; (f) capacity-building facilities for research purposes; (g) other forms of benefit consistent with the principles set out in this Declaration.

Guideline 2 of CIOMS’ International Ethical Guidelines for Health-related Research involving Humans provides a similar specification in terms of provision of the fruits of research to the population or community where the research was carried out, most especially for research conducted in low-resource settings.^109^ Then, on Guideline 20, it stipulates the applicability of Guideline 2 for research during disaster and disease outbreaks.^110^

During the Covid-19 pandemic, we see these principles tested again and again. There were moments filled with high spirits and solidarity. The sequencing of the genome of SARS-CoV-2, as well as the identification of relevant proteins and enzymes, happened at lightning speed because of spontaneous collaboration between universities from different countries.^111^ We also saw how public funds were earmarked Covid-19 research and therapy development.^112^^,^^113^^,^^114^ At the same time, we saw how public funds were used to secure advanced orders of potential Covid-19 vaccines originally researched using public funds^115^ and how pharmaceutical companies played hardball with the public for Covid-19 testing kits.^116^ There were real reasons to be worried, most especially if pharmaceutical companies continue to be granted free rein in pricing, which usually means paying “a small fortune” for new interventions.^117^

To address potential access concerns brought about by patent market exclusivity,^118^ Costa Rica spearheaded what is now the WHO Solidarity Call for a Covid-19 Technology Access Pool.^119^ Specifically, this is a call for “key stakeholders and the global community to voluntarily pool knowledge, intellectual property and data necessary for Covid-19”.^120^ It was this same spirit of solidarity that spurred the sequencing of the genome of the SARS-CoV-2 and it is this same spirit of pooling, collaboration, and sharing of benefits that provides hope for timely and equitable access to much needed interventions.^121^ Research ethics has provided this foundation, as we saw above, and it remains research ethics’ task to ensure that Covid-19 drug discovery and development take this course. To date, 38 countries have signed the WHO Solidarity Call, which countries such as the US and the UK have yet to become signatories.

## The role of research ethics in research on and with those most vulnerated by the Covid19-pandemic: the case of elderly people in nursing homes

Older people in nursing homes are among the most vulnerable in contemporary society. This has become very clear in several countries during the Covid19-pandemic. In May 2020 a report from Bergen (one of the largest cities in Norway) revealed that 87% of the city’s Covid-19 deaths stemmed from patients living in nursing homes.^122^ And in Sweden nursing home residents account for nearly half of deaths linked to Covid-19,^123^ while future estimates in England are that more than half of coronavirus-related deaths will affect people living in care homes.^124^

Research on how to prevent deaths in nursing homes during a pandemic, and ways of minimizing the risk of contagion represent issues of great ethical and health political urgency. There are, however, several research ethical challenges related to the inclusion of nursing home residents in research projects because the pandemic has rendered them victims of *additional* vulnerability.^125^ The more vulnerable a research participant is, the greater the risk of causing harm. Furthermore, the inherent asymmetric power relation between the researcher and his/her patients fuels power-imbalance, about which we as researchers should be ever more concerned, Many older and vulnerable nursing home patients, many of whom either lack the competency to consent, or are verbally deficient. The principle of consent is one of the most basic research principles. The UN Human Rights Committee notes that special protection is warranted when including persons not capable of giving valid consent.^126^ In some cases a next of kin may consent on behalf of the patient, but since we cannot be sure whether this consent reflects the wishes of the patient him/herself, this poses a problem. We should therefore be aware of both verbal and non-verbal signs and reactions from the participants’ signs of discomfort and resistance throughout the research process. Here we will argue that researchers need a moral sensitivity, not only to avoid physical harm or risks, but also to avoid psychological and social damage.^127^ It is not sufficient to conform to “procedural ethics” at the onset of a research-project or to gain/secure approval from a Research Ethical Committee. Moral sensitivity in research may be even more important during pandemics, when research protocols seem to be “rushed” and misgivings ignored. In addition to moral sensitivity as researchers, we may also argue for a kind of ethical calmness or what Guillemin and Gillam refer to as “ethical reflexivity”^128^ throughout the research process.

## Concluding remarks

The arguments presented above suggest that the role of research ethics in pandemic times is exceptionally important, but not only in the sense of deviating from hard won core ethical and epistemological principles in the wake of WWII. On the contrary, perhaps more than ever, it is vital to restate the importance of human dignity, human rights and fundamental freedoms as the normative bedrock on which medical research involving human subjects should rely. There is no alternative pathway for research ethics that is viable; returning to the core values and principles enshrined in the Universal Declaration of Human Rights is urgently needed. We therefore share the views expressed by the European Group on Ethics in Science and New Technologies in a Statement on the Covid-19 pandemic^129^:

It is natural in these circumstances of deep uncertainty to focus on immediate action and speed of measures. This must not, however, lead to a continuous suspension of rights and liberties. We therefore call for vigilance about the necessity, evidence, proportionality of any policy and technological intervention that, even temporarily, suspends fundamental rights. Consideration needs to be given to the immediate and lasting impacts that such measures have on our societies.

## Footnotes

1 For a differentiation between three different forms of uncertainty, see next paragraph below and Table 1.

**Table 1:** Covid-19 and three forms of uncertainty (risk, strict uncertainty and ignorance)

*Risk*^a,b,c,d^ (known outcomes and known probability distributions)	Test accuracy (sensitivity, specificity, predictive values) for the various tests in different contexts Effects and side effects of new treatments Prevalence of disease The risk of healthcare workers versus the risk of non-essential workers testing positive for COVID-19
*Strict uncertainty*^e,f,g,h^ (known outcomes and unknown probability distributions)	Basic reproduction number (R) Case fatality rate/infection fatality rate The precise interval during which an individual with SARS-CoV-2 infection can transmit infection The pathogenic effect of the SARS-CoV-2 in different age groups The extent to which transmission occurs from a-symptomatic or pre-symptomatic subjects and how much it contributes to the pandemic Whether all infected patients mount a protective immune response and how long any protective effect will last Reinfection How long SARS-CoV-2 can persist on surfaces Whether pre-existing immune responses impact the risk or the severity of COVID-19 and whether they will influence SARS-CoV-2 vaccine responses Long-term sequelae and late-stage consequences of COVID-19
*Ignorance*^i,j,k,l^ (unknown outcomes and unknown probability distributions)	Mutations Treatment options Unexpected obstacles to vaccine development and production

^a^Goldstein and Burstyn (2020)

^b^Sethuraman et al. (2020)

^c^Mutambudzi et al. (2020)

^d^McIntosch et al. (2020)

^e^Hofmann (2020)

^f^McIntosch et al. (2020)

^g^Yelin et al. (2020)

^h^European Group in Ethics in Science and Technology (2020)

^i^McIntosch et al. (2020)

^j^Hofmann (2020)

^k^Yelin et al. (2020)

^l^Kalil (2020)

2 BMA (2001, p. 205).

3 BMA (2001, p. 205).

4 BMA (2001, p. 210).

5 Stoeklé and Hérve (2020).

6 Hellmann et al. (2020).

7 Outka (2020).

8 Wynne (1992), Rørtveit and Strand (2001), Nielsen and Sørensen (2017) and Hofmann (2020).

9 https://www.ohchr.org/en/udhr/pages/udhrindex.aspx.

10 United States vs. Karl Brandt, et al. (Case No. 1) Trials of War Criminals before the Nuremberg Military Tribunals under Control Council Law No. 10. Superintendent of Documents, U. S. Government Printing Office Washington D.C. Volume I (Nuremberg Military Tribunals) pp. 181–182.

11 Callaway (2020), Eyal (2020), Eyal et al. (2020), Jamrozik and Selgelid (2020a, b), Plotkin and Caplan (2020), Schaefer et al. (2020), Shah et al. (2020), Singer and Chappell (2020), WHO (2020) and Wolemonwu (2020).

12 Singer and Chappell (2020).

13 WHO (2020).

14 Shah et al. (2020).

15 Jamrozik and Selgelid (2020a).

16 United States vs. Karl Brandt, et al. (Case No. 1) Trials of War Criminals before the Nuremberg Military Tribunals under Control Council Law No. 10. Superintendent of Documents, U. S. Government Printing Office Washington D.C. Volume I (Nuremberg Military Tribunals) p. 182.

17 United Nations International Covenant on Civil and Political Rights. Adopted and opened for signature, ratification and accession by General Assembly resolution 2200A (XXI) of 16 December 1966, entry into force 23 March 1976, in accordance with Article 49.

18 UN Doc A/2929 pp. 87–88. United Nations General Assembly Tenth Session. Draft International Covenants on Human Rights. Annotation Prepared by the Secretary General. 1 July 1955.

19 Ibid p. 88.

20 UN Human Rights Committee (HRC), CCPR General Comment No. 20: Article 7 (Prohibition of Torture, or Other Cruel, Inhuman or Degrading Treatment or Punishment), 10 March 1992, para. 3, available at: https://www.refworld.org/docid/453883fb0.html [accessed 5 June 2020].

21 The Academy of Medical Sciences (2018, p. 3).

22 The International Alliance for Biological Standardization (2019), Jamrozik and Selgelid (2020a), Eyal (2020).

23 Shah et al. (2020).

24 Singer and Chappell (2020).

25 WHO (2020) and Jamrozik and Selgelid (2020a).

26 Eyal (2020), Eyal et al. (2020), Jamrozik and Selgelid (2020a, b), Plotkin and Caplan (2020), Schaefer et al. (2020), Shah et al. (2020), Singer and Chappell (2020), WHO (2020).

27 In a recent paper reviewing and synthesizing available evidence on asymptomatic SARS-CoV-2 infection, subclinical lung abnormalities was found even among asymptomatic persons (For this see Oran 2020).

28 This additional form of uncertainty is admitted by Jamrozik and Selgelid (2020a, p. 4).

29 Jamrozik and Selgelid (2020a, p. 4) briefly address the question whether “the permissibility of high-risk human challenge studies” would increase if researchers used themselves as research participants, but they warn against this, and notably for the reason that “clinical and research staff might feel pressure to participate”.

30 WHO (2016).

31 WHO (2016, pp. 8–9).

32 WHO (2016, p. 9).

33 Lynch (2020).

34 Cohen (2020)—For this, see also Guarino and Johnson (2020).

35 Elliott (2020).

36 Macklin (2020).

37 Elliott (2020).

38 Ibid.

39 Ibid.

40 Jamrozik and Selgelid (2020a). This concern is also raised by Deming et al. (2020), and by Schaefer et al. (2020).

41 Shah et al. (2020).

42 Shah et al. (2020), WHO (2020, note 27, p. 13).

43 Ward et al. (1958) and Krugman and Giles (1973).

44 Goodman and McElligott (2003, p. 125).

45 Krugman (1971, pp. 966–967).

46 These figures are mentioned in Southam’s letter to Mandel dated July 5, 1964. For this letter, see next note.

47 Letter from Chester M. Southam, M.D., to Emanuel Mandel, M.D.—July 5, 1963. In: Katz (1972, pp. 10–11).

48 Arras (2008, p. 75).

49 This presentation of the interview with Southam, is taken from E. Langer’s article. Human Experimentation: Cancer Studies at Sloan-Kettering Stir Public Debate on Medical Ethics. Science, New Series, Vol. 143, No. 3606 (Feb. 7, 1964), pp. 551–553.

50 «Direct evidence» was Southam’s own wording in the letter to Mandel.

51 Southam in his letter to Mandel.

52 Ibid.

53 A CHI-study in 197 healthy volunteers contributed to the development and licencing of the Live Oral Cholera Vaccine CVD 103-HgR study in 1993. For this, see: Roestenberg et al. (2018, p. 4).

54 In 2015, the first malaria vaccine allegedly achieved through the use of CHI-studies gained EMA approval. For this, see Roestenberg et al. (2018, p. 4). For this, see also: European Medical Agency: https://www.ema.europa.eu/en/documents/medicine-outside-eu/mosquirix-summary-public_en.pdf.

55 For such a study, see: Graham et al. (2004).

56 Miller and Grady (2001, p. 1032).

57 Ibid., p. 1032.

58 CIOMS (2016, p. 10).

59 The International Alliance for Biological Standardization (2019, p. 88).

60 Ibid., p. 88. For a similar verdict concerning a Zika CHI-trial, see Recommendation 2 in Shah et al. (2017, p. 27): “Whether a Zika virus human challenge trial has sufficient social value to proceed depends on the reasons for doing it and whether there are alternative ways to obtain the information. The most compelling rationale for conducting a Zika virus human challenge trial, given the risks and uncertainty, would be if field trials were prohibitively difficult to conduct in light of a waning epidemic. This rationale is not currently met, but it could come to pass in the future. Another valuable reason to conduct a challenge trial would be to accelerate the development of a vaccine that could prevent congenital Zika infection. This rationale must be accompanied with strong evidence that results from a Zika virus human challenge trial would be used by stakeholders (e.g., indication from regulatory agencies that finding a correlate would speed up the licensing of a vaccine). The committee did not hear sufficient evidence that this rationale is currently met. Finally, using a challenge trial solely to learn about the pathogenesis and natural history of Zika infection is unlikely to justify the risk involved given the alternative ways to obtain similar information”.

61 Jamrozik and Selgelid (2020a, p. 4).

62 WHO (2020, p. 14).

63 Goodman and McElligott (2003, p. 125).

64 Shah et al. (2020, p. 2). For the scientific merit argument, see also Bambery et al. (2020, pp. 93–94), Shah et al. (2017, pp. 20–22), Jamrozik and Selgelid (2020b, pp. 602–603), and Wolemonwu (2020, p. 2).

65 Krugman (1971, p. 966).

66 Shah et al. (2020, p. 2).

67 Singer and Chappell (2020). For this argument, see also Eyal et al. (2020, p. 1754), Menikoff (2020, p. 81), Schaefer et al. (2020).

68 Singer and Chappell (2020), Shah et al. (2020, p. 1), WHO (2020, p. 1), Jamrozik and Selgelid (2020a, p. 1).

69 The International Alliance for Biological Standardization (2019, p. 86): “Performing CHI is a way to learn and test, while minimizing the number of subjects”.

70 Krugman (1971).

71 Jamrozik and Selgelid (2020a, p. 3).

72 WHO (2020, p. 9). For this argument, see also Eyal et al. (2020, pp. 1754–1755), and Eyal (2020, p. 26).

73 Krugman (1971, p. 967).

74 Shah et al. (2020, p. 2).

75 WHO (2020, p. 9). Also Eyal (2020, p. 26) highlights the importance of Covid-19-CHI-trial participants having “access to standard-of-care life-sustaining treatments”.

76 Krugman (1971, p. 967).

77 WHO (2020, p. 8). For the immunity argument, see also Eyal (2020, p. 30).

78 Jamrozik and Selgelid (2020a, p. 3). A slightly different version of this argument reads thus: “In short, if researchers conducting challenge trials act as recommended, admittedly, the probability of getting infected would remain larger inside a challenge trial than either outside any trial or in a standard efficacy trial; but the probability of death or disability is likely to be much smaller inside a challenge trial than in these alternative scenarios. Overall, a × b could be smaller for any individual inside the challenge trial than either outside any trial or in a standard efficacy trial. What the individual would lose in the probability of averting infection (with that probability rising) she could gain in better protection from death” (Eyal 2020, p. 27).

79 Krugman (1971, p. 967): informed consent by proxy; Shah et al. (2020, p. 3): “Robust consent”; Jamrozik and Selgelid (2020a, p. 4): “proper” or “adequate consent”; WHO, 220, p. 15: “SARS-CoV-2 challenge studies must involve rigorous informed consent”. For this, see also Bambery et al. (2020, pp. 97–98), Eyal (2020, p. 29), Plotkin and Caplan 2020, p. 3987), Schaefer et al. (2020), and Wolemonwu (2020, p. 3).

80 Shah et al. (2020, p. 3): «Sites should be selected for sound scientific reasons while avoiding especially vulnerable populations”; WHO (2020, p. 13): “Those whose background risk is high as a result of social injustice should be excluded from participation because their inclusion could be considered unethical exploitation (i.e., taking advantage of those who have already been wrongly disadvantaged. Any prospective participants who could reasonably be perceived to be vulnerable in other ways that would undermine their consent or put them at greater risk (for example as a result of the mental health strain of inpatient isolation during the study) should also be excluded”.

81 Jamrozik and Selgelid (2020b, p. 11).

82 https://www.cirp.org/library/ethics/helsinki/.

83 Singer and Chappell (2020).

84 London and Kimmelman (2020, pp. 476–477).

85 Ibid., p. 476.

86 Ibid, p. 477.

87 Doroshow et al. (2020).

88 Ioannidis (2020).

89 Retraction Watch. Retracted coronavirus (COVID-19) papers. https://retractionwatch.com/retracted-coronavirus-covid-19-papers/.

90 Mehra et al. (2020a).

91 Mehra et al. (2020b).

92 University of Oxford. Dexamethasone reduces death in hospitalised patients with severe respiratory complications of COVID-19. June 16, 2020. Accessible at: https://www.ox.ac.uk/news/2020-06-16-dexamethasone-reduces-death-hospitalised-patients-severe-respiratory-complications. On October 16, 2020, WHO reported that “dexamethasone is the only effective drug for coronavirus”. This information is accesible at: https://www.aa.com.tr/en/latest-on-coronavirus-outbreak/who-dexamethasone-only-effective-drug-for-coronavirus/2009114.

93 The expression “doing science by press release” we have borrowed from an interview about the study with Dr. George Anesi, director of the Medical Critical Care Bioresponse Team at the Hospital of the University of Pennsylvania. Accessible at: https://www.nbcnews.com/health/health-news/science-press-release-doctors-view-covid-19-drug-results-excitement-n1231183.

94 NBC News. Doctors view dexamethasone results on COVID-19 with excitement and skepticism. Accessible at: https://www.nbcnews.com/health/health-news/science-press-release-doctors-view-covid-19-drug-results-excitement-n1231183.

95 Macklin (2020).

96 Acosta et al. (2015).

97 Langmuir (1979, p. 660).

98 Sencer and Millar (2006).

99 Reference is here made to: Department of Health and Human services. Annex 11: Pandemic influenza response and preparedness plan. Washington: The Department; 2003 Aug 26.

100 FDA Announces Two Drugs Given ‘Compassionate Use’ Status in Treating COVID-19, 19.03, 2020. Accessible at: https://www.pharmacytimes.com/news/fda-announces-two-drugs-approved-for-compassionate-use-in-treating-covid-19; and, FDA is allowing two drugs to be used for ‘compassionate use’ to treat the coronavirus. Here's what that means. Accessible at: https://www.businessinsider.com/chloroquine-remdesivir-compassionate-use-coronavirus-what-it-means-2020-3?op=1&r=US&IR=T.

101 US FDA. Expanded Access. https://www.fda.gov/news-events/public-health-focus/expanded-access. For this, see also, European Medicines Agency. Compassionate use. Accessible at: https://www.ema.europa.eu/en/human-regulatory/research-development/compassionate-use.

102 Borysowski et al. (2017).

103 Ibid.

104 Ibid.

105 https://www.centerwatch.com/articles/12702-new-mit-study-puts-clinical-research-success-rate-at-14-percent.

106 Angus (2020).

107 WMA (2013).

108 UNESCO (2005).

109 ‘CIOMS’ stands for Council for International Organizations of Medical Sciences. For this, see CIOMS (2016).

110 Ibid.

111 Editorial in Nature. Everyone wins when patents are pooled. Nature, 2020; 581: 240.

112 AJ Impact/Europen Union (2020).

113 NOVAVAX (2020).

114 CNBC (2020a).

115 CNBC (2020b).

116 DutchNews.nl (2020).

117 Lazarus (2020).

118 Hoen (2020).

119 COVID-19 technology access pool, 2020. https://www.who.int/emergencies/diseases/novel-coronavirus-2019/global-research-on-novel-coronavirus-2019-ncov/covid-19-technology-access-pool.

120 Ibid.

121 Emanuel et al. (2020).

122 Bergens tidende: Accessible at: https://www.bt.no/nyheter/lokalt/i/70vxd4/26-av-30-doede-var-sykehjemsbeboere.

123 BBC News. Accessible at: https://www.bbc.com/news/world-europe-52704836.

124 The Guardian. Accessible at: https://www.theguardian.com/society/2020/jun/07/more-than-half-of-englands-coronavirus-related-deaths-will-be-people-from-care-homes.

125 The differentiation between vulnerability and being vulnerated is important to distinguish between on the one hand persistent and on the other variable forms of vulnerability (Solbakk 2011, pp. 228–238). The persistent form of vulnerability we all share, is part of the human condition, while the second form of vulnerability is context-dependent, in the sense that some people because of disease, poverty, lack of freedom etc. are vulnerated, i.e. harmed or wounded. This distinction points to the need for a differentiation between at least two distinct regimes of protection. Firstly, a human rights-based regime aimed at protecting persistent or universal vulnerability. This regime requires *negative* action on the part of the State, in the sense that its responsibility is to guarantee basic liberties by securing a just social order that gives equal protection to the vulnerability of each citizen. These protective measures are, however, in need of being supplemented by additional measures of protection—of *affirmative* action—to cope with accidental states and situations when human vulnerability is no longer intact. “Fallen vulnerability” is a metaphor that has been suggested to designate such situations (Kottow 2004, p. 281).

126 UN Human Rights Committee (HRC), *CCPR General Comment No. 20: Article 7 (Prohibition of Torture, or Other Cruel, Inhuman or Degrading Treatment or Punishment)*, 10 March 1992, para. 7, available at: https://www.refworld.org/docid/453883fb0.html [accessed 5 June 2020].

127 Heggestad et al. (2013).

128 Guillemin and Gillam (2004). Guillemin, M., & Gillam, L. (2004). Ethics, Reflexivity, and “Ethically Important Moments” in Research. Qualitative Inquiry, 10(2), 261–280. 10.1177/1077800403262360.

129 European Group in Ethics in Science and Technology. Statement on European Solidarity and the Protection of Fundamental Rights in the COVID-19 Pandemic, April 2, 2020. Accessible at: https://ec.europa.eu/info/sites/info/files/research_and_innovation/ege/ec_rtd_ege-statement-covid-19.pdf.
